# WorldFengur - the studbook of origin for the Icelandic horse

**DOI:** 10.1186/1751-0147-53-S1-S5

**Published:** 2011-06-20

**Authors:** Jón Baldur Lorange

**Affiliations:** 1 107 Reykjavík, Iceland

## Abstract

WorldFengur is the database that contains and functions as the studbook of origin of the Icelandic horse. Only pure-bred Icelandic horses, whose ancestry can be traced back to Iceland entirely, may be registered into WorldFengur. The WorldFengur project is a joint effort by the FAIC (Farmers Association of Iceland) and FEIF (International Federation of Icelandic Horse Associations) to construct an official and central database on horses of Icelandic origin located all over the world. It is used in this capacity in 19 countries so far; the number of data stored in the WorldFengur database has increased continuously. The database itself has developed tremendously since it was established in 2001; it includes information on horses’ pedigrees and offspring, as well as results of breeding assessments and sports competitions, owners, breeders, breeding prediction values (BLUP), colours, microchip numbers, health records, DNA profiles for checking ancestries and much more. The key words in its development are common solutions to common challenges internationally. The requirements to fulfill both national and international regulations, such as the latest EU directive on the identification of equidae - no 504/2008/EU -, have increased in recent years and the WorldFengur project continuously endeavours to stay in line with these developments.

## What is WorldFengur?

*WorldFengur* (WF) is the international database containing the studbook of origin of the Icelandic horses. The name means “the world’s benefit”. WorldFengur was opened formally in August 2001 at the World Championships for Icelandic horses in Austria. WF contains information on Icelandic horses world-wide. It is a joint effort by the FAIC, Farmers Association of Iceland, and FEIF, International Federation of Icelandic Horse Associations, in order to develop an official, global and central database on horses of Icelandic origin located all over the world. 19 countries are full members or associate members of FEIF. Icelandic horses from non-FEIF countries such as Poland, Spain, Australia and Hungary, are also registered into WF, provided their national registration guarantees them being pure-bred. One of the main objectives of the WF project is to enable the calculation of global breeding evaluations of Icelandic horses, based on their sharing the same genepool, by using measureable and comparable data stored in WF from as many countries as possible [1]. Another of the main objectives of the WF project is to provide a common IT solution to common challenges for all approved breeding associations that keep studbooks for Icelandic horses. IT technology has provided unique opportunities to reach this goal. We have created access to all relevant and real-time information world-wide in one place, and have provided additional knowledge by cross-linking information in comprehensive databases.

**Figure 1:**
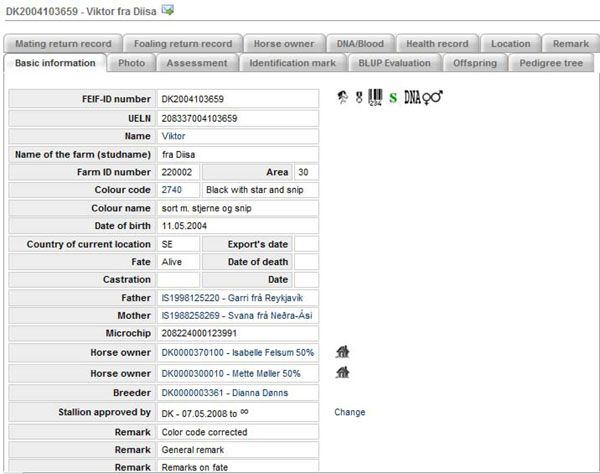
Basic information on a horse

In WF, one finds a wide-range of information about 354,035 pure-bred Icelandic horses all over the world (see Table 1), both alive and deceased, such as pedigrees, offspring, breeding assessments, sport competitions, owners, breeders, breeding prediction values (BLUP), colours, microchip numbers, health records, DNA profiles and more. A total of 220,168 horses of those 354,035 are registered to be alive (62%). *Identification documents* (horse passports, article 21 of Chapter VII of the EC 504/2008 directive) of Icelandic horses in Iceland, Denmark, Sweden, Faroe Islands and Great-Britain are issued by the official breeding associations in those countries using WF software, which is in accordance with the EU directive no (EC) 504/2008. Through WF, it is possible to keep track of all movements of animals within and across borders, provided of course that these are registered into WF. Figure 1 shows the *Basic information page* for a stallion. Information on all the offspring of the stallion can be found on the tab *Offspring*, information on all assessments can be found on the tab *Assessment*, etc. The icons to the right of the FEIF-ID numbers on Figure 1 indicate that the relevant information exists in WF; for example DNA♀♂ means that DNA markers for this horse and its parents are available in WF and nothing refutes the pedigree of the horse.

**Table 1:** Information about 354,035 pure-bred Icelandic horses all over the world

Country	Total number of horses ***located*** in country	Percentage of total number of horses	Horses alive	Percentage of total number of horses alive	Less than 1 year old horses
Iceland	205,704	58.103%	99,996	45.418%	6,881
Denmark	38,782	10.954%	31,473	14.295%	2,369
Germany	31,820	8.988%	25,598	11.627%	2,427
Sweden	29,823	8.424%	24,715	11.226%	1,441
Norway	12,456	3.518%	11,428	5.191%	391
Netherlands	8,905	2.515%	7,240	3.288%	264
United States	5,041	1.424%	4,162	1.890%	109
Austria	3,230	0.912%	2,723	1.237%	136
Finland	3,114	0.880%	2,641	1.200%	89
Switzerland	3,350	0.946%	2,284	1.037%	63
Canada	2,098	0.593%	1,671	0.759%	33
Unknown	4,385	1.239%	1,658	0.753%	
Belgium	1,535	0.434%	1,415	0.643%	57
France	1,270	0.359%	1,099	0.499%	11
Great-Britain	1,126	0.318%	929	0.422%	32
Italy	362	0.102%	294	0.134%	7
Faroe Islands	293	0.083%	219	0.099%	2
Luxembourg	190	0.054%	174	0.079%	2
Slovenia	168	0.047%	159	0.072%	3
Australia	105	0.030%	86	0.039%	1
New Zealand	78	0.022%	72	0.033%	5
Ireland	40	0.011%	35	0.016%	
Hungary	27	0.008%	26	0.012%	
Romania	20	0.006%	20	0.009%	
Greenland	45	0.013%	19	0.009%	
Poland	11	0.003%	9	0.004%	
Mexico	7	0.002%	7	0.003%	7
Estonia	5	0.001%	5	0.002%	
Liechtenstein	3	0.001%	3	0.001%	
Russia	2	0.001%	2	0.001%	
Czech Republic	2	0.001%	2	0.001%	
Croatia	2	0.001%	2	0.001%	
Lithuania	34	0.010%	1		
Portugal	2	0.001%	1		
					
**Total:**	**354.035**	100%	**220.168**	100%	**14.330**

Source: WF [3], 25.11.2010

The key is to create powerful, flexible and secure access control, and a fast 24-hours service. The success of the WF project is based on the 3 Cs: *Connectivity*, *Coordination and Cooperation*. It connects people world-wide, coordinates their work and creates successful cooperation.

## Technical information

WF is a web-based program (Java Server Pages, JSP) connected to an Oracle™ database which stores all its data. The hardware is based on a client browser, web server, Oracle Application Server and Oracle Database System version 10g. The development tool is JavaDeveloper™ from Oracle. The communication is 3-tier layer. First, there is a presentation layer based on websites, then there is a communication layer in accordance with the TCP/IP standard, and finally, there is an Oracle Application Server (OAS) which communicates exclusively with the database.

## Flexible and secure access control

As mentioned before, the key is access control. The FAIC and FEIF have agreed on a set of standardised rules and procedures concerning access rights to WF. This is a part of the agreement between both parties that was signed in 2000. MAST, *The Icelandic Food and Veterinary Authority*, is in charge of access rights and registration rules on animal welfare issues (such as diseases and medication) in accordance with currently valid laws and regulations. Each DNA laboratory has exclusive rights to register its own DNA testing results, as well as having access to results registered by other approved laboratories in the WF/DNA project. Each country's member association provides a list of persons who are given access at different levels. All members of the 19 FEIF associations - currently appr. 60,000 - can get free subscriber access (i.e., read-only access) to WF through their associations. In the past 12 months, 9,000 of those members made use of their access to WF. Access to the system depends on the users' rights. For instance, a user with Danish registrar access is only allowed to register and change basic information on horses born in Denmark, and has limited access to registering data for horses located in Denmark. One user may have more than one function or role combined in his or her access, e.g. both registrar's and judge's access. The most common user groups in WF are FEIF registrars, national registrars, national judges, national veterinarians, laboratory employees and read-only subscribers. WorldFengur keeps track of the history of registration of and changes made to data; this is important in order to guarantee the system's transparency regarding the origin of data. Changes of most important data are done through registering remarks, in order to make the data history more transparent. These remarks are restricted to the registrar that made them, so only he or she can change or delete them.

## Health records

**Figure 2:**
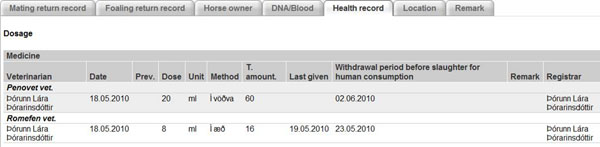
Information on medicinal treatment for a horse

All practicing veterinarians on Iceland are given access to WF. Access is controlled by MAST. In WF, all diseases and medicinal treatments for individual horses, or groups of horses must be registered in accordance with national as well as EU laws. Veterinary registration of medicinal treatment in WF started in March 2010 (Figure 2). By using WF it is guaranteed that all treated horses are pure-bred and that they have been micro-chipped and registered into WF both with a UELN number - the Universal Equine Life Number, (see the UELN website, http://www.ueln.net) - and with a FEIF-ID number, which is a unique and universal identification number for pure-bred Icelandic horses. MAST thus has access to a powerful surveillance tool through WF to overview health records for all horses. The health record function has only been implemented in Iceland so far.

## The WF/DNA Project

**Figure 3:**
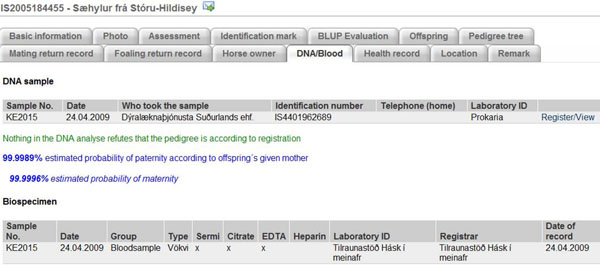
DNA genetic result for a horse (proof of parentage)

Today, proof of parentage by DNA is the most effective tool to guarantee that all registered horses in WF are of Icelandic origin. For this purpose, international cooperation was established between laboratories to gather DNA testing results from various countries. When DNA markers (genotypes) have been imported from data files or have been registered manually in WF, a specially designed program within WF runs an automatic test comparing the horse’s markers with the DNA markers of its parents to confirm the ancestry, provided the parents’ markers are available in the database. Then, as can be seen on Figure 3, WF gives a notification if nothing in the DNA analyse refutes that the pedigree is in accordance with the ancestry registered. Also, WF calculates the probability of paternity according to offspring’s given mother. All DNA testing results must be performed in accordance with the ISAG standard (International Society of Animal Genetics). At present, WF imports DNA genotyping results from the following laboratories: Matís (Prokaria) in Iceland, Swedish University of Agricultural Science (SLU), Certagen in Germany and Blodtypelabatoriet for heste (The Blood type laboratory for horses) in Denmark. Furthermore, DNA results from UC Davis in USA, Van Haeringen Laboratorium b.v. in Netherlands, Genlab Niini in Finland and GeneControl in Germany have been entered as well, though these laboratories are not direct partners in the WF/DNA project.

## Conclusions and outlook

WF is a good example of how IT can be used to connect people and data around the world. The comprehensive data in WF enhances knowledge that can be used for breeding Icelandic horses. Since WF opened 10 years ago it has strengthened international cooperation and coordination within the Icelandic horse community. The so-called positive spill-over effect is very much valid in the WF project. Functions already implemented in WF create strong incentives for developing new functions in different sectors. The result is more integration, coordination and cooperation. In the beginning, the main aim was to build an international database to collect data on pedigrees of as many Icelandic horses as possible, which could be used to calculate a breeding prediction evaluation. In 2007, Jens Iversen, who was then president of FEIF said in an interview in *Bændablaðið* (the Icelandic Farmers Newspaper): “WorldFengur is the most important tool that FEIF has to keep FEIF’s work going. No other discipline in equestrianism has access to such comprehensive information as the Icelandic horse.” [2]. Step by step new functions and responsibilities, such as DNA, Sport results and Health information, are added to WF which connects the Icelandic horse enthusiasts more and more all over the world. For further information, see the http://www.worldfengur.com[3].
